# Use of Fundus Autofluorescence Combined with Optical Coherence Tomography for Diagnose of Geographic Atrophy in Age-Related Macular Degeneration

**Published:** 2019-10-01

**Authors:** Nathalie Massamba, Alexandre Sellam, Nathalie Butel, Dimitra Skondra, Violaine Caillaux, Bahram Bodaghi

**Affiliations:** 1 Department of Ophthalmology, DHU: Handicaps and Vision Pitié Salpetrière University Hospital, University of Pierre Marie Curie, Paris, France; 2 Department of Ophthalmology, Visual Sciences University Hospital of Chicago, Illinois, USA.; 3 J. Terry Ernest Ocular Imaging Center, University of Chicago, Chicago, Illinois, USA

**Keywords:** Macular Degeneration, Choroidal Neovascularization, Tomography, Optical Coherence, Vascular Endothelial Growth Factors, Sensitivity and Specificity

## Abstract

The aim of this study was to demonstrate the sensitivity of Optical coherence tomography (OCT) in detection of geographic atrophy (GA) secondary to exudative age related macular degeneration (AMD). In this retrospective case series study 77 patients (53% female, with mean ± standard deviation [SD] of 82.6±9.3 years) with 97 eyes (45 OS [left eyes]/52 OD [right eyes]) were included. This was a retrospective review of the charts of patients who presented with exudative AMD at the Pitié Salpetrière Hospital, Paris, France, between December 2016 and August 2017 that received intravitreal injections of anti-vascular endothelial growth factor (anti-VEGF) therapies. At baseline, following biomicroscopy examination, multimodal imaging was performed including, fluorescein angiography (FA), fundus auto-fluorescence (FAF), spectral domain optical coherence tomography (SD-OCT) and indocyanine green angiography (ICGA). During the follow-up, SD-OCT with/without FAF and FA were performed for each patient at 6, 12 and 18 months. For investigation of the prevalence of GA in eyes undergoing intravitreal injections with anti-VEGF therapy, FAF and SD-OCT images were qualitatively reviewed by four independent observers (two graders per group). Kappa coefficient of Cohen was calculated to determine agreement between the graders. The kappa coefficient of Cohen, for inter-rater agreement in the evaluation of FAF images was 0.468, indicating a moderate agreement between the first and second raters. Thus, the sensitivity and specificity of FAF for the diagnosis of GA were 70% and 57%, respectively. If atrophy was assessed with SD-OCT image analysis, the kappa coefficient for inter-rater agreement was 0.846, implying an acceptable agreement between both readers. The sensitivity and specificity of SD-OCT were 93% and 58% respectively. In conclusion, SD-OCT image analysis was more sensitive than FAF for identifying GA in patients treated for exudative AMD.

## INTRODUCTION

Age-related macular degeneration (AMD) is the main reason for blindness after 50 years of age [1] and is a phenotypical heterogeneous disease that includes dry (atrophic) and wet (exudative) forms. The wet form is characterized by an unusual choroidal neovascularization (CNV) in the macula [2]. The Anti-vascular endothelial growth factor (Anti-VEGF) has been successful in improving visual prognosis of patients with neovascular age-related macular degeneration (nAMD) and other wet (exudative) retinal Conditions by intravitreal injection [3]. There are three anti-VEGF agents currently used globally including Ranibizumab (Lucentis; Genentech, Inc., South San Francisco, CA, USA), Aflibercept (Eylea; Regeneron Pharmaceuticals, Inc., Tarrytown, NY, USA) and Bevacizumab (Avastin; Genentech, Inc.). In France, however, the regulatory authorities prevent the off-label usage of Bevacizumab in ophthalmology procedure [4], and hence only Ranibizumab and Aflibercept are available. Moreover, both these drugs are administered for all patients in France and choosing between these drugs is independent of economic considerations or patients’ affiliation with insurance agencies. The safe usage and frequency of Bevacizumab and Ranibizumab treatment of macular degeneration related to nAMD was uncertain, hence, the purpose of the IVAN clinical trial in the United Kingdom was to undertake the hindrance of VEGF in CNV [5]. In the USA, the most popular trial, Comparison of Age-related macular degeneration Treatments Trial (CATT) was designed as a substitute [6]. The 2-year CATT [7] reports portrayed the differences and similarities between drugs and the process of treatment to develop a new geographic atrophy (GA) in the eye under study during the follow-up. There were no differences among the drugs in IVAN trial compared with the CATT and IVAN data combined. Moreover, our detailed study showed a high and steady increase in the risk of getting a new GA with injections in monthly basis compared to intermittent treatment. It was a concern that any benefit from monthly treatment may not be maintained in the longer term [8].

**Figure 1:**
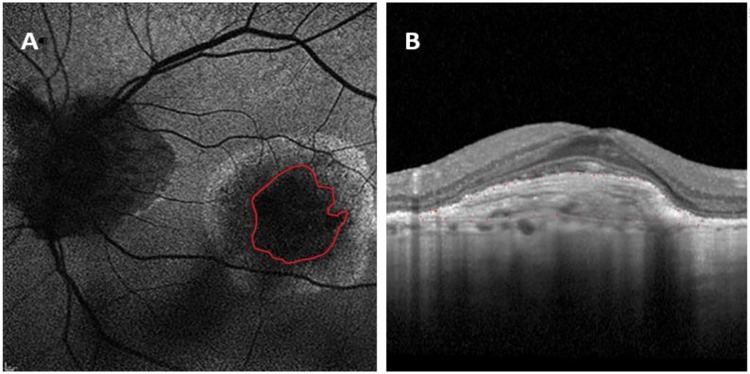
Patient 1 – Left eye of a 65-year-old female patient with exudative Age related macular degeneration (AMD). A: Fundus auto-fluorescence (FAF) showed a large macular lesion hypo auto-fluorescence surrounded by a halo hyper auto-fluorescence. B: Spectral-domain optical coherence tomography (SD-OCT), showed a homogeneous lesion localized above the RPE. The hypo auto-fluorescence lesion is a fibrosis without retinal pigment epithelium (RPE) atrophy lesion

**Figure 2:**
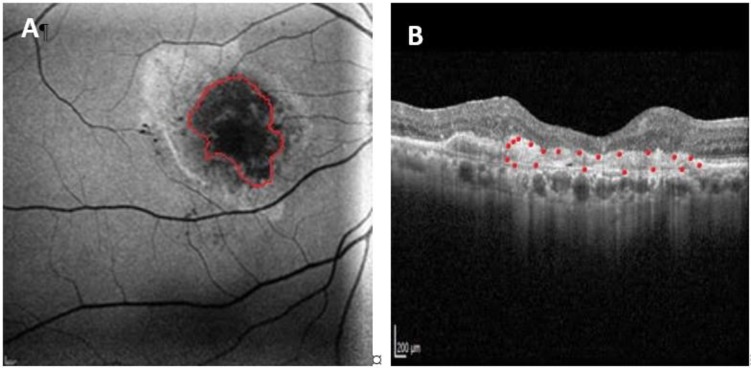
Patient 2- Right eye; a 74-year-old female patient presented with exudative age related macular degeneration (AMD) and received 21 intravitreal injection of anti-vascular endothelial growth factor (Anti-VEGF). A: Fundus auto-fluorescence (FAF) showed a hypo auto-fluorescence lesion on the macula surrounding by a hyper auto-fluorescence in the temporal superior of the lesion. B: Spectral-domain optical coherence tomography (SD-OCT) showed homogeneous lesion localized above the RPE with a thinning of the pigment epithelium, and the outer retina layer, next to the fovea. The lesion corresponding to a fibrosis associated with atrophy of the retinal pigment epithelium (RPE).

**Figure 3:**
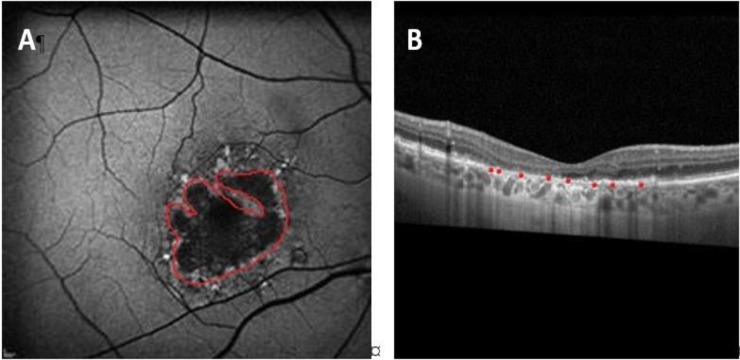
Patient 3- A 70-year-old male patient with exudative age related macular degeneration (AMD) on the right eye who received 5 intravitreal injections of anti-vascular endothelial growth factor (Anti-VEGF). Initially, he had an atrophic lesion before the treatment. A: Fundus auto-fluorescence (FAF) showed a large macular lesion hypo auto-fluorescence surrounded by a hyper auto-fluorescence halo. B: Spectral-domain optical coherence tomography (SD-OCT), of the same eye showed thinning of the retina pigment epithelium (RPE), and the outer retina layer, next to the fovea. The lesion is atrophic despite their different outliner, all lesions are hypo auto-fluorescence, which makes easier the diagnosis of geographic atrophy (GA), but the SD - OCT confirms precisely the difference between the atrophic lesion (Fig 3) versus Fibrosis associated with GA (Fig 2) and the fibrosis (Fig 1)

Geographic atrophy secondary to AMD has the key characteristics of progressive augmentation of these spots of retinal atrophy, which includes the outer retina, choroid and retinal pigment epithelium (RPE) [2]. For prevention of GA development, there is no treatment at the moment. In the recent times, Fundus Autofluorescence (FAF) has been used to follow GA progression in a multicenter natural history study entitled “Fundus Autofluorescence Imaging in Age-Related Macular Degeneration (FAM)” [9, 10]. The FAF imaging allows topographical mapping of distribution of lipofuscin (LF) in the monolayer RPE, in the outer retina and sub-neurosensory space [11, 12]. This imaging provides additional information that cannot be obtained using other modalities such as fluorescein angiography (FA), or spectral-domain optical coherence tomography (SD-OCT) and fundus photography. Color fundus photography is widely used for the measurement of the size of GA areas in the epidemiologic and natural history studies [4, 6]. Nevertheless, color fundus photography has several shortcomings in the determination of GA primarily to interpatient variations of fundus pigmentation and variations of drusen appearance. The FAF is predominantly used in everyday clinical practice. It is a noninvasive imaging method and depends on retinal fluorophores LF, which accumulates with aging RPE post-phagocytosis [7, 8]. The FAF uses an excitation wavelength of 488 nanometer (nm) and bandwidth for emission in the range of 500-700 nm. The imaging has been successful especially for various aspects in GA secondary to AMD. In atrophic areas of the macula, there is a loss of RPE cells due to loss of intrinsic LF fluorophores. The GA areas exhibit low to dark FAF signals (zero), with a huge difference among the area of atrophy and perilesional retina [13]. Thus, using SD-OCT imaging, choroidal signal enhancement due to loss of absorbing pigment and thinning of the outer retinal layers such as the outer nuclear layer is evident at the site of GA while these findings are often corresponded to a lower FAF signal. SD-OCT showed also a hypo intensity for lesions above RPE, as fibro-glial lesion. In Seven-up study, [14] evaluation of the presence of GA was based on the FAF. However, both the GA and fibrosis are hypo-autofluorescence lesions which might be challenging to be differentiated (Figures 1 and 2). Thus, the main goal of our study was to understand and differentiate detection of GA assessed by SD-OCT or FAF in patients treated for an exudative AMD developing secondary GA (Figure 3).

## METHODS

A retrospective review was performed on patients’ charts presented with exudative AMD at the Pitié Salpetrière Hospital, Paris, France, between December 2016 and August 2017 who received anti-VEGF by intravitreal injection. At baseline, following biomicroscopy examination, multimodal imaging was performed including FA, FAF, Spectralis HRA+SD-OCT (Heidelberg Engineering, Heidelberg, Germany) and indocyanine green angiography (ICGA). While the follow-up treatment period, SD-OCT with or without FAF and FA were performed in each patient at 6, 12 and 18 months. The FAF and SD-OCT images were qualitatively reviewed by four independent observers (two readers per group) to determine whether GA was present. In accordance with the tenets of the Declaration of Helsinki, a written informed consent was obtained from each participant. For data review, the approval of French Society of Ophthalmology Ethics Committee was taken. The inclusion criteria were; 1) patients with exudative AMD as evaluated with fundus biomicroscopic and FA, FAF, ICGA at baseline, 2) treatment with anti-VEGF intravitreal injection (at least 6 injections during the follow-up period).

Only, patients who developed GA during anti-VEGF treatment were included in the study. Patients with CNV because of causes other than AMD such as inflammatory disease or other retinopathies (including diabetic retinopathy and retinal venous occlusion) were excluded. The FAF images were received using a short wavelength (488 nm). This imaging modality has been shown to provide an adequate assessment of atrophy and currently considered the best available tool to define areas of RPE loss [15]. As per our clinical standards, at first treatment consisted of a series of three intravitreal injections of anti-VEGF performed by a retinal specialist using established techniques. This protocol comprised of three initial injections (monthly installments) based on Pro ReNata regiment (PRN) [16] decision to withdraw or not in the advancement of visual acuity (VA) and presence or absence of sub-fovea fluid as seen by OCT. After the initial treatment, for every subsequent month, patients underwent a lengthy medical and ocular analysis, procedure of corrected distance visual acuity (CDVA) with the help of the Early Treatment Diabetic Retinopathy Study (ETDRS) charts and ophthalmic assessment, which consists of a slit-lamp bio-microscopic examination, intraocular pressure (IOP) measurement, fundus bio-microscopic, SD-OCT examination, FAF and FA. So as to investigate the prevalence of GA in eyes that received intravitreal injections of anti-VEGF, FAF images from the last follow-up exam was qualitatively reviewed by two independent observers (AS and NB) (appearing as hypofluorescent regions on FAF). In cases of discordance between the two graders, a third senior grader (NM) opinion was required. Similarly, two readers (NB and VC) analyzed SD-OCT images and in cases of discrepancy between them a third senior grader (NM) resolved it. Statistical analysis was performed using SPSS version 22.0 (IBM Corp, Amont NY, USA). All values were expressed as mean ± Standard deviation (SD). Data analysis was performed using Wilcoxon signed rank to analyze the SD values between different batches. A P value less than 0.05 was considered statistically significant. Interclass correlation coefficient (ICC) was used to assess agreement of measurements between the readers.

## RESULTS

Initially, 119 eyes of 97 consecutive patients (55 females and 42 males) were selected. Of these, 22 eyes of 20 patients were excluded (six eyes of 6 patients imaged by TOPCON, 12 eyes of 12 patients did not have FAF and 4 eyes of 2 patients had low quality of images). Overall, 97 eyes (45 left eye [OS] versus 52 right eye [OD]) of 77 patients with mean ± SD age of 82.6± 9.3 years (range, 94-67) were considered in the experimental analysis (Table 1). Inter-agreement of FA between the readers were 0.464 and 0.846 for SD-OCT. The sensitivity and specificity of the FAF analysis for the diagnosis of GA were 70% and 57%, respectively, suggesting a modest rate for both and, the kappa coefficient for inter-rater agreement was 0.46, implying moderate agreement among readers. However, when GA was assessed using SD-OCT image analysis, the kappa coefficient for inter-rater agreement was 0.8, implying as an almost perfect agreement between both readers. Even though, the sensitivity was 93%, indicating a low rate of false negatives; however, the specificity was 58%, suggesting a modest rate of false positives with SD-OCT (Table 2). These results showed that SD-OCT image analysis is more sensitive and accurate than FAF for identifying GA in patients being treated for exudative AMD. Using both methods of analysis may significantly improve the accurate diagnosis of GA in patients with AMD.

**Table 1:** Demographic Characteristics of the Study Participants

Characteristics of the Participants (Total: 97 eyes; 77 patients)		
Laterality of Subjects’ Eye (n)	52 OD	45 OS
Gender (n)	41 M	36 F
Age (Y) Mean ± SD	M=79.7±11.07	F=82.6±9.3

Abbreviations: n: number; OD: right eye; OS: left eye; M: male; F: female; SD: standard deviation.

**Table 2:** Strength of Agreement Using SD-OCT and FAF Between Two Readers

	Clinical Features of SD-OCT and FAF				
Imaging Methods	**Sensitivity**	**Specificity**	**ICC**	**Strength of Agreement (Altman’s Kappa Benchmark Scale)**	
FAF	70%	57%	0.464(0.434-0.506)	Moderate	
SD-OCT	93%	58%	0.846(0.811-0.918)	Very Good	

Abbreviations: %: percentage; ICC: The Intraclass Correlation Coefficient; SD-OCT: spectral domain optical coherence tomography; FAF: Fundus autofluorescence.

## DISCUSSION

In this cross-sectional study, we investigated GA in AMD eyes previously treated with anti-VEGF and with absence of neovascular activity (no sub-retinal hemorrhage, a sign of fluid in or under the retina and no treatment for at least 6 months). We found that eyes with evidence of fibrovascular scar are characterized by a worse structural and functional impairment. The GA is normally, specified as an acute circumscribed surface of RPE atrophy through which choroidal vessels can be viewed. Notably, we used the FAF and SD-OCT imaging to distinguish eyes with excessive increase in the fibrous component, which characterizes a fibrocellular scar phenotype.

AMD is one of the most common reasons for blindness which is irreversible in elderly in highly developed countries [17] and includes two forms of wet (exudative) and dry (atrophic) AMD. RPE dysfunction disrupts both photoreceptors and choroidal vasculature; this tissue disruption leads to tissue atrophy and neovascularization. Histologically, the exudative lesion is a complex structure composed of different constituents, including blood vessels, macrophages, myofibroblasts and fibroblasts [18, 19]. Although the pathogenic sequence leading to macular fibrosis in neovascular AMD is still not well understood, Ishikawa et al. thought that it could be based on a complex tissue repair mechanism [20]. However, Dry (atrophic) AMD has the characteristics of a progressive course to degeneration of photoreceptors and RPE. It is identified by a loss of the outer neurosensory retina, the thinning of RPE and the choriocapillaritis. Hence, both atrophic and advance of exudative AMD forms lead to damage and death of photoreceptors. As noted above FAF imaging permits topographical connection between distribution of LF in the RPE and mapping of other fluorophores that might happen with the disease in the outer retina, and the sub neuro-sensory space [21, 22]. Due to absence of RPE cells and reduction of intrinsic LF fluorophores, atrophic areas in patients with GA exhibit a severely reduced signal (dark). Therefore, in advanced form of exudative AMD, changes due to photoreceptors loss, increase of melanin and presence of scar could probably block the fluorophores induced by the FAF (Dark). 

The main goal of our study was to distinguish detection of GA assessed by SD-OCT or FAF in patients treated for an exudative AMD developing secondary GA. In our retrospectively study, we reviewed charts of patients with exudative AMD who received intravitreal anti-VEGF injections during follow-up examinations. Patients with FAF and SD-OCT images taken in the follow-up process were considered in the analysis to identify GA. We found that sensitivity and specificity of the FAF analysis for the diagnosis of GA were 70% and 57%, respectively, suggesting a modest rate for both. However, when atrophy was assessed using SD-OCT image analysis, the kappa coefficient for an inter-rater agreement was 0.8, implying that the agreement between both readers was almost perfect. 

Several studies have demonstrated the apoptotic loss of the RPE as a necessary last stage symptom of severe AMD [23]. The authors also confirmed that ARPE-19 cells, a widely accepted RPE cell line, displayed necrotic features consisting of poly (ADP-ribose) polymerase-1 (PARP-1) activated by hydrogen peroxide (H2O2). They reported that apoptosis-inducing factor (AIF) - independent PARP - 1 - dependent necrosis creates a primary mechanism of RPE cell loss, thus cause a retinal degeneration in case of a severe AMD. Moreover, Du. J et al. [24] demonstrated in their recent study that reductive carboxylation capacity in RPE overshoots all other tested cells, not limited to retina, glial cells, neural tissue and a cancer cell line. Loss of reductive carboxylation damages redox balance and dramatically heightens RPE sensitivity to oxidative damage, proposing that shortcomings of reductive carboxylation might stimulate RPE cell loss. By analyzing their study, we concluded that the glial cells had also a reductive carboxylation and lead to cell death [24]. Therefore, we can conclude that RPE is the predilection site for cell death in retinal pathologies [25], particularly severe and dry AMD, but the elements in this process to death are independent. Without speculated, it is considered that the cell death would cause hypo-fluorescence phenome visualized in FAF in atrophy form or advanced AMD. With SD-OCT, the atrophy form reveals choroidal hyperreflectivity behind the RPE reduction of the photoreceptor layer. Both the quintessential findings in GA and fibrosis appear as a compact hyper-reflective component located in the sub-RPE or the sub-retinal compartment, and it was demonstrated that it could be more frequent than RPE atrophy [26]. CATT study group [7] (two years) used both the FA and the SD- OCT in the follow-up period, because the authors found that there might be numerous anatomical observations identified only on SD-OCT. The authors believed that the fluorescence blocked on FA corresponded to a particular area of hypofluorescence, which is contiguous to the CNV and generally, not influenced by visible hemorrhage, pigmentation or other conditions observed on the color photography. The wedge of fluorescence could represent a fibrosis after advance stage of the nAMD or atrophic form [7]. In the context of differential diagnosis of atrophy in FAF, including scotoma, metamorphosis, adult-onset vitelliform dystrophy is a common adult disease. This frequent pathology presents itself in FA like CNV type 2. Bhakhri R et al. [27] in their case report demonstrated the importance of SD-OCT and FAF in differential diagnosis. The authors described FAF atrophy as a hypoautofluorescent central lesion surrounded by a hyper autofluorescent, which could be an accumulation of photoreceptor debris, pigment granules (hypo autofluorescent) and LF (hyper autofluorescent ). In SD-OCT, the diagnosis already showed an empty space between the RPE and photoreceptors indicative of a sensory retinal detachment**.**

Choroidal melanocytic is a benign tumor caused by proliferation of the melanocytes normally found in the uvea. Singh AD et al. [28], have shown the importance of the FAF combined with SD-OCT in clinical practice sets and for the detection of prognostic factors. Authors compared detection rates of drusen and subretinal fluid by fundus photographs with high-definition Fourier-domain optical coherence tomography (FD-OCT) and that of orange pigment by FAF in patients with indeterminate choroidal melanocytic lesion (IML) (categorized as small tumor). Their findings indicated that changes in small melanoma range from chronic changes such as drusen and thinning of the retina, subretinal fluid that overlaps with those observed in nevus and melanoma. We believed that both exams are noninvasive and important, but they should be coupled for a better diagnosis. Our result corroborated to Holz et al. [29], who demonstrated the advantage of combining SD-OCT and FAF confocal scanning laser ophthalmoscope imaging for GA assessments.

We agree with Chen Q et al. [30], who improved visualization technique of GA in SD-OCT images and a new semi-automated GA segmentation method. They emphasized the advantage of developing GA segmentation and quantification techniques in SD-OCT images over other modalities. The authors attested that the images in FAF are the result of an in-depth integration, superimposing different retinal structures. These images may overestimate or underestimate certain pathologies. Hence, pairing FAF with the SD-OCT and certifying it as the gold standard in GA has been matter of interest. 

Our study had several limitations, primarily due to the retrospective nature of imaging analysis, monocentric aspect and lack of measurement and analysis of atrophy on FAF at baseline and during the follow-up. Analysis on FAF was not used because we did not prove the need of measurement and VA measurement between fibrosis lesion and GA undergoing AMD. Further, prospective and multicentric investigations are necessary to reconfirm our hypothesis. Studies of a small number of subjects can be easy to conduct by analyzing patient records and images by ophthalmologists specialist in retinal imaging. Therefore, an obvious strength is that the research can be addressed in a relatively short period. Obtaining ethical and institutional approval is easier in small studies than large multicenter studies. It is often accurate to test a new research hypothesis with a small number and generate a hypothesis, rather than a large multicentric confirmation study. Thus, as, being a retina specialist specialized in AMD, our discussions led us to conclude that the FAF alone was not enough strong for the diagnosis of GA. Hence, SD-OCT combined with FAF was a better approach for diagnosing and monitoring patients with atrophic and exudative AMD. Another strong point was to have a Spectralis HRA, which had FAF and SD-OCT on the same imaging device.

## CONCLUSIONS

SD-OCT image analysis is more sensitive than FAF for identifying GA in patients treated for exudative AMD.

## DISCLOSURE

Ethical issues have been completely observed by the authors. All named authors meet the International Committee of Medical Journal Editors (ICMJE) criteria for authorship of this manuscript, take responsibility for the integrity of the work as a whole, and have given final approval for the version to be published. No conflict of interest has been presented.

## Funding/Support

None.
